# Neuroendocrine Tumors of the Large Intestine: Clinicopathological Features and Predictive Factors of Lymph Node Metastasis

**DOI:** 10.3389/fonc.2016.00173

**Published:** 2016-07-18

**Authors:** Motohiro Kojima, Koji Ikeda, Norio Saito, Naoki Sakuyama, Kenichi Koushi, Shingo Kawano, Toshiaki Watanabe, Kenichi Sugihara, Masaaki Ito, Atsushi Ochiai

**Affiliations:** ^1^Division of Pathology, Exploratory Oncology Research and Clinical Trial Center, National Cancer Center, Kashiwa, Japan; ^2^Division of Surgical Oncology, National Cancer Center Hospital East, Kashiwa, Japan; ^3^Advanced Clinical Research of Cancer, Juntendo University Graduate School of Medicine, Tokyo, Japan; ^4^Division of Surgical Oncology, The University of Tokyo, Tokyo, Japan; ^5^Division of Surgical Oncology, Tokyo Medical and Dental University, Tokyo, Japan

**Keywords:** neuroendocrine tumor, carcinoid tumor, WHO classification 2010, lymph node metastasis, rectum, colon

## Abstract

A new histological classification of neuroendocrine tumors (NETs) was established in WHO 2010. ENET and NCCN proposed treatment algorithms for colorectal NET. Retrospective study of NET of the large intestine (colorectal and appendiceal NET) was performed among institutions allied with the Japanese Society for Cancer of the Colon and Rectum, and 760 neuroendocrine tumors from 2001 to 2011 were re-assessed using WHO 2010 criteria to elucidate the clinicopathological features of NET in the large intestine. Next, the clinicopathological relationship with lymph node metastasis was analyzed to predict lymph node metastasis in locally resected rectal NET. The primary site was rectum in 718/760 cases (94.5%), colon in 30/760 cases (3.9%), and appendix in 12/760 cases (1.6%). Patients were predominantly men (61.6%) with a mean age of 58.7 years. Tumor size was <10 mm in 65.4% of cases. Proportions of NET G1, G2, G3, and mixed adeno-neuroendocrine carcinoma (MANEC) were 88.4, 6.3, 3.9, and 1.3%, respectively. Of the 760 tumors, 468 were locally resected, and 292 were surgically resected with lymph node dissection. Rectal NET showed a higher proportion of NET G1, and colonic and appendiceal NET was more commonly G3 and MANEC. Of the 292 surgically resected cases, 233 NET G1 and G2 located in the rectum were used for the prediction of lymph node metastasis. Lymphatic and blood vessel invasion were independent predictive factors of lymph node metastasis. NET G2 cases showed more frequent lymph node metastasis than that seen in NET G1 cases, but this was not an independent predictor of lymph node metastasis. Of the 98 surgically resected cases <10 mm in size, we found 9 cases with lymph node metastasis (9.2%). All cases were NET G1, and eight of the nine cases were positive either for lymphatic invasion or blood vessel invasion. Using the WHO classification, we found NET in the large intestine showed a tumor-site-dependent variety of histological and clinicopathological features. Risk of lymph node metastasis in rectal NET was confirmed even in lesions smaller than 10 mm. Concordant assessment of vascular invasion will be required to estimate lymph node metastasis in small lesions.

## Introduction

A new histological classification of neuroendocrine tumors (NETs) was determined in the WHO 2010 classification, and the UICC TNM classification seventh edition or ENET classification proposed the staging of gastrointestinal NET (1–3). NETs arise from the whole organ, and gastrointestinal NET was traditionally divided by the embryological origin of the foregut, midgut, and hindgut. Midgut and hindgut NET arise in the large intestine and the variable histologic features of NET in this organ could complicate identification even when using identical WHO 2010 classifications. Furthermore, large clinicopathological data sets of NET based on the WHO 2010 classification are not available, and the utility of the WHO 2010 classification as a prognostic factor is not well established in NET of the large intestine. Recently, clinical guidelines for gastrointestinal NET were also proposed by NCCN and ENET (4, 5). However, the indication for local excision or endoscopic resection in small lesions is not fully determined (6). Similar to early colorectal adenocarcinoma, the clinicopathological association between colorectal NET and lymph node status in surgically resected cases has been studied to predict lymph node metastasis (7, 8). In this study, retrospective assessment of NET of the large intestine (colorectal and appendiceal NET) was performed among the 68 Japanese institutions allied with the Japanese Society for Cancer of the Colon and Rectum (JSCCR) to clarify the clinicopathological features of NET in the large intestine using WHO 2010 criteria. Next, using surgically resected rectal NET G1 and G2, the clinicopathological relationship with lymph node metastasis was analyzed to assess the current therapeutic algorithm.

## Materials and Methods

Pathological re-evaluations of NET according to the WHO 2010 criteria were performed in cases with NET of the large intestine removed from 2001 to 2011 in the 68 institutions of the JSCCR. The collected clinicopathological information was patient age, sex, tumor size, tumor location, pathological diagnosis based on WHO 2010 criteria, depth of tumor invasion, mitotic index [<2/10 high power fields (HPF), 2–20/10 HPF, or >20/10 HPF], Ki-67 labeling index (≤2, 3–20, or >20%), and resection method (surgical, endoscopic, or other). Information of lymph node metastasis (present or absent) was collected in all surgically resected cases, and cases underwent radical surgery after the local resection were included in resected cases. These data were registered into a Web system developed by the JSCCR. Clinical outcome or prognosis was not investigated in this study. Clinicopathological features of NET in the large intestine were evaluated, and compared among rectal, colonic, and appendiceal NET, and the association between clinicopathological features and lymph node metastasis was assessed using surgically resected rectal NET G1 and G2 to estimate risk factors of lymph node metastasis in locally resected tumors. This study was approved by the review board in the JSCCR and National Cancer Center (2014-332).

### Statistical Analysis

All continuous data were described as mean and SD in this study. As therapeutic strategy of NET in large intestine is divided by tumor size of 10 and 20 mm, case number and frequency of the tumors <10, ≥10 and <20, and ≥20 mm were calculated. Comparison of clinicopathological features among rectal, colonic, and appendiceal NET was performed using the χ^2^-test and Student’s *t*-test.

Clinicopathological factors associated with lymph node metastasis in rectal NET G1 and G2 were assessed using χ^2^-test and Student’s *t*-test, and multiple logistic regression analyses were performed to identify independent predictive factors of synchronous lymph node metastasis. A probability value of <0.01 was regarded as statistically significant in this study.

## Results

In this study, 760 cases from 68 institutions were entered and clinicopathological features were evaluated (Table 1). Of the 760 tumors, 718 (94.5%) were located in the rectum, 30 (3.9%) were in the colon, and 12 (1.6%) were in the appendix. In total, 461 (60.7%) patients were men and 299 (39.3%) were women, with a mean age of 58.7 years. The frequencies of NET G1, G2, NEC (G3), and mixed adeno-neuroendocrine carcinoma (MANEC) were 672 (88.4%), 48 (6.3%), 30 (3.9%), and 10 (1.3%), respectively. Of the 760 NET, 468 were locally resected, and 292 were surgically resected with lymph node dissection, which included cases who underwent radial surgery after the local resection. As for tumor size, the mean size was 11.7 mm, and 497 tumors (65.4%) were smaller than 10 mm. Most tumors were limited to the mucosa or submucosa (657 cases, 86.4%), showed mitosis in <2/10 HPF (694 cases, 91.3%), and had a Ki-67 index of ≤2% (675 cases, 88.8%). Lymphatic vessel invasion and blood vessel invasion were found in 136 (17.9%) and 173 (22.8%) cases, respectively. Lymph node metastasis was found in 108 cases of 292 surgically resected cases (37.0%). As for clinicopathologic differences among colonic, rectal, and appendiceal NETs, patient ages were different among them, significantly (*P* < 0.01), and were in the order of colonic (68.6 ± 11.9 years), rectal (58.6 ± 12.4 years), and appendiceal NET (44.7 ± 22.6 years). Distribution of histological grade was also different between rectal NET and others. Compared with colonic and appendiceal NET, rectal NET was predominantly NET G1 (656 cases, 91.4%). However, colonic NET included more NET G3/NEC (14 cases, 46.7%) and MANEC (6 cases, 20.0%), and appendiceal NET included more NET G3/NEC (7 cases, 58.3%). Most of the local resections were performed in rectal NET (467/468 cases, 99.8%). Tumor sizes were 9.7 ± 12.3 mm for rectal NET, 51.3 ± 37.8 mm for colonic NET, and 28.8 ± 36.2 mm for appendiceal NET; rectal NETs were significantly smaller than colonic and appendiceal NET (*P* < 0.01). The distribution of tumor depth is also different between rectal NET and others, and tumor depth in rectal NET was limited to the mucosa or submucosa in 684 cases (95.3%), while over half of colonic and appendiceal NET invaded the muscular layer or deeper (*P* < 0.01). Concordant with WHO 2010 criteria, cases with low mitotic and Ki-67 indices were seen more often in rectal NET than in others. Lymphatic vessel and blood vessel invasions were more frequently seen in colonic NET than in rectal NET. Also, lymph node metastasis in surgically resected cases was seen more frequently in colonic NET than in others, significantly (*P* < 0.01).

**Table 1:** **Clinicopathological features of colorectal neuroendocrine tumor based on WHO 2010 criteria**.

		Total cases	Rectal NET	Colonic NET	Appendiceal NET	Rectal vs. colonic (*P* value)	Rectal vs. appendiceal (*P* value)	Colonic vs. appendiceal (*P* value)
No. of cases		760	718 (94.5%)	30 (3.9%)	12 (1.6%)			
Age (years)	Mean ± SD	58.7 ± 12.8	58.6 ± 12.4	68.6 ± 11.7	44.7 ± 22.6	>0.01	>0.01	>0.01
Sex	Men	461 (60.7%)	435 (60.4%)	20 (66.6%)	6 (50.0%)	0.50	0.46	0.31
	Women	299 (39.3%)	283 (39.4%)	10 (33.3%)	6 (50.0%)			
WHO 2010 criteria	NET G1	672 (88.4%)	656 (91.4%)	9 (30.0%)	3 (25.0%)	>0.01	>0.01	0.18
	NET G2	48 (6.3%)	44 (6.1%)	1 (3.3%)	2 (16.7%)			
	NET G3/NEC	30 (3.9%)	14 (2.0%)	14 (46.7%)	7 (58.3%)			
	MANEC	10 (1.3%)	4 (0.6%)	6 (20.0%)	0 (0%)			
Resection	Surgically	292 (38.4%)	251 (35.0%)	29 (96.7%)	12 (100%)	>0.01	>0.01	0.52
	Locally	468 (61.6%)	467 (65.0%)	1 (3.3%)	0 (0%)			
Tumor size	Mean ± SD	11.7 ± 16.9	9.7 ± 12.3	51.3 ± 37.8	28.8 ± 36.2	>0.01	>0.01	0.09
	<10 mm	497 (65.4%)	490 (68.2%)	4 (13.3%)	3 (25.0%)	>0.01	>0.01	0.66
	≥10 mm, <20 mm	180 (23.7%)	176 (24.5%)	3 (10.0%)	1 (8.3%)			
	≥20 mm	83 (10.9%)	52 (7.2%)	23 (76.7%)	8 (66.7%)			
Tumor depth	Limited to mucosa (%)	32 (4.2%)	32 (4.5%)	0 (0%)	0 (0%)	>0.01	>0.01	0.25
	Invades submucosa (%)	625 (82.2%)	652 (90.8%)	4 (13.3%)	1 (8.3%)			
	Invades muscularis propria (%)	42 (5.5%)	39 (5.4%)	2 (6.7%)	1 (8.3%)			
	Invades into subserosa or non-peritonealized pericolonic or perirectal tissue (%)	38 (5.0%)	21 (2.9%)	9 (30.0%)	8 (66.7%)			
	Invades peritoneum or other organs (%)	23 (3.0%)	6 (0.8%)	15 (50.0%)	2 (16.7%)			
Mitotic index (/10HPF)	<2 (%)	694 (91.3%)	676 (94.2%)	9 (30.0%)	9 (75.0%)	>0.01	0.02	0.012
	≤2, ≤20 (%)	31 (4.1%)	25 (3.5%)	4 (13.3%)	2 (16.7%)			
	>20 (%)	35 (4.6%)	17 (2.4%)	17 (56.7%)	1 (8.3%)			
Ki-67 index (%)	≤2 (%)	675 (88.8%)	659 (91.8%)	9 (30.0%)	7 (58.3%)	>0.01	>0.01	0.03
	<2, ≤20 (%)	48 (6.3%)	42 (5.8%)	3 (10.0%)	3 (25.0%)			
	>20 (%)	37 (4.9%)	17 (2.4%)	18 (60.0%)	2 (16.7%)			
Lymphatic vessel invasion	Absent (%)	624 (82.1%)	607 (84.5%)	9 (30.0%)	8 (66.7%)	>0.01	>0.01	0.18
	Present (%)	136 (17.9%)	111 (15.5%)	21 (70.0%)	8 (66.7%)			
Blood vessel invasion	Absent (%)	587 (77.2%)	572 (79.7%)	7 (23.3%)	8 (66.7%)	>0.01	0.27	>0.01
	Present (%)	173 (22.8%)	146 (20.3%)	23 (76.7%)	4 (33.3%)			
Lymph node metastasis	Absent (% in surgical cases)	184 (63.0%)	166 (66.1%)	8 (27.6%)	10 (83.3%)	>0.01	0.22	>0.01
	Present (% in surgical cases)	108 (37.0%)	85 (33.9%)	21 (72.4%)	2 (16.7%)			

*Clinicopathologic features of colorectal neuroendocrine tumors removed from a Japanese cohort from 2001 to 2011 and retrospectively classified using the WHO 2010 criteria*.

*NEC, neuroendocrine carcinoma; NETs, neuroendocrine tumors; MANEC, mixed adeno-neuroendocrine carcinoma*.

Next, we investigated the risk factor of synchronous lymph node metastasis, especially in small rectal NET G1 and G2. At first, we evaluated the association between clinicopathological features and lymph node metastasis using all 233 surgically resected rectal NET G1 and G2. Clinicopatholgoical features are shown in Table 2, and lymph node metastasis was found in 70 cases (30.0%) in these cases. Table 3 shows the association between clinicopathological features and lymph node metastasis. NET G2, large tumor size, tumor invasion into the muscularis propria or deeper, high mitotic index, high Ki-67 index, lymphatic vessel invasion, and blood vessel invasion were associated with lymph node metastasis in univariate analysis. Furthermore, lymphatic and blood vessel invasion were independent predictors of lymph node metastasis in multivariate analysis. Although NET G2 cases showed more lymph node metastasis than that seen in NET G1 cases, that was not an independent predictor of lymph node metastasis in rectal NET. Table 4 (A) shows the association between clinicopathological features and lymph node metastasis in surgically resected rectal NET G1 and G2 <20 mm. Tumor size, lymphatic invasion, and blood vessel invasion were independent predictors of lymph node metastasis. NET G2 cases tended to show more frequent lymph node metastasis, but this difference was not statistically significant. Of 489 rectal NET G1 and G2 <10 mm, 98 were surgically resected, and 391 were locally resected. The differences between them are shown in Table 5. Surgically resected cases were larger and showed more frequent lymphatic vessel and blood vessel invasion than that seen with locally resected cases, and lymph node metastasis was seen in 9 cases (9.2%) of 98 surgically resected cases. A clinicopathological comparison between cases with and without lymph node metastasis is shown in Table 4 (B). Of the clinicopathological features, although not an independent risk factor in multivariate analysis, blood vessel invasion was found more frequently in cases with lymph node metastasis in the univariate analysis. The clinicopathological features of nine cases with lymph node metastasis are shown in Table 6. All nine cases were NET G1, and the tumor depth was limited to the mucosa or submucosa. Importantly, eight of the nine cases showed either lymphatic or blood vessel invasion.

**Table 2:** **Clinicopathological features of surgically resected rectal NET G1 and G2 based on WHO 2010 criteria**.

Total cases		233
Age (years)	Mean ± SD	57.7 ± 12.0
Sex	Men	133 (57.1%)
	Women	100 (42.9%)
WHO 2010 criteria	NET G1	201 (86.3%)
	NET G2	32 (13.7%)
Tumor size (mm)	Mean ± SD	12.9 ± 12.6
Location	Upper rectum	37 (15.9%)
	Lower rectum	196 (84.1%)
Tumor depth	Limited to mucosa	6 (2.6%)
	Invades submucosa	183 (78.5%)
	Invades muscularis propria	34 (14.6%)
	Invades into subserosa or non-peritonealized pericolonic or perirectal tissue	10 (4.3%)
	Invades peritoneum or other organs	0 (0%)
Mitotic index (/10HPF)	<2	212 (91.0%)
	≤2, ≤20	21 (9.0%)
Ki-67 index (%)	≤2	202 (86.7%)
	<2, ≤20	31 (13.3%)
Lymphatic vessel invasion	Absent	165 (70.8%)
	Present	68 (29.2%)
Blood vessel invasion	Absent	149 (63.9%)
	Present	84 (36.1%)
Lymph node metastasis	Absent	163 (70.0%)
	Present	70 (30.0%)

*Study population described in Table 1*.

*NETs, neuroendocrine tumors*.

**Table 3:** **Association between clinicopathological features and lymph node metastasis in surgically resected rectal NET G1 and G2 Lymph node metastasis**.

		Positive (70 cases)	Negative (163 cases)	*P* value (univariate analysis)	*P* value (multivariate analysis)
Age (years)	Range (mean ± SD)	56.3 ± 11.3	58.3 ± 12.2	0.24	
Sex	Men	39	94	0.78	
	Women	31	69		
Location	Upper rectum	7	30	0.11	
	Lower rectum	63	133		
WHO 2010 criteria	NET G1	54	147	*P* < 0.01	0.89
	NET G2	16	16		
Tumor size (mm)	Range (mean ± SD)	16.6 ± 9.9	11.3 ± 13.3	*P* < 0.01	0.08
Tumor depth	Limited to mucosa or submucosa	45	144	*P* < 0.01	0.05
	Invades muscularis propria or deeper	25	19		
Mitotic index (/10HPF)	<2	58	154	*P* < 0.01	
	≤2, ≤20	12	9		
Ki-67 index (%)	≤2	16	15	*P* < 0.01	
	<2, ≤20	54	148		
Lymphatic vessel invasion	Absent	32	133	*P* < 0.01	*P* < 0.01
	Present	38	30		
Blood vessel invasion	Absent	28	121	*P* < 0.01	*P* < 0.01
	Present	42	42		

*Study population described in Table 1*.

*NETs, neuroendocrine tumors*.

**Table 4:** **Association between clinicopathological features and lymph node metastasis in surgically resected rectal NET G1 and G2 <20 and <10 mm**.

(A) Association between clinicopathological features and lymph node metastasis in surgically resected rectal NET G1 and G2 <20 mm					(B) Association between clinicopathological features and lymph node metastasis in surgically resected rectal NET G1 and G2 <10 mm				
*N* = 200	Lymph node metastasis		*P* value (univariate analysis)	*P* value (multivariate analysis)	*N* = 98	Lymph node metastasis		*P* value (univariate analysis)	*P* value (multivariate analysis)
	Positive (53 cases)	Negative (147 cases)				Positive (9 cases)	Negative (89 cases)		
Age (years; mean ± SD)	55.5 ± 11.6	58.2 ± 12.3	N.S		Age (years; mean ± SD)	57.1 ± 8.8	57.7 ± 12.3	N.S	
Sex					Sex				
Men	30	85	N.S		Men	5	51	N.S	
Women	23	62			Women	4	38		
Location					Location				
Upper rectum	7	27	N.S		Upper rectum	0	21	N.S	
Lower rectum	46	120			Lower rectum	9	68		
WHO 2010 criteria					WHO 2010 criteria				
NET G1	43	133	0.07		NET G1	9	85	N.S	
NET G2	10	14			NET G2	0	4		
Tumor size (mm; mean ± SD)	12.5 ± 3.5	8.5 ± 3.8	*P* < 0.01	*P* < 0.01	Tumor size (mm; mean ± SD)	7.2 ± 1.6	6.1 ± 2.0	N.S	
Tumor depth					Tumor depth			N.S	
Limited to mucosa or submucosa	40	131	0.02		Limited to mucosa or submucosa	9	86		
Invades muscularis propria or deeper	13	16			Invades muscularis propria or deeper	0	3		
Mitotic index (/10HPF)					Mitotic index (/10HPF)				
<2	45	139	0.03		<2	9	86	N.S	
≤2, ≤20	8	8			≤2, ≤20	0	3		
Ki-67 index (%)					Ki-67 index (%)			N.S	
≤2	42	134	0.02		≤2	9	87		
<2, ≤20	11	13			<2, ≤20	0	2		
Lymphatic vessel invasion					Lymphatic vessel invasion			0.02	
Absent	26	121	*P* < 0.01	*P* < 0.01	Absent	5	76		
Present	27	26			Present	4	13		
Blood vessel invasion			*P* < 0.01	*P* < 0.01	Blood vessel invasion			<0.01	=0.01
Absent	22	109			Absent	3	68		
Present	31	38			Present	6	21		

*Study population described in Table 1*.

*NETs, neuroendocrine tumors*.

**Table 5:** **Clinicopathological difference between locally resected and surgically resected rectal NET G1 and G2 <10 mm**.

		Treatment		
n = 489		Local resection	Surgical resection	*P* value (univariate analysis)
		391	98	
Age (years)	Range (mean ± SD)	58.3 ± 12.7	57.6 ± 12.0	0.62
Sex	Men	238	56	0.50
	Women	153	42	
Location	Upper rectum	73	21	0.54
	Lower rectum	318	77	
WHO 2010 criteria	NET G1	382	94	0.07
	NET G2	9	4	
Tumor size (mm)	Range (mean ± SD)	6.2 ± 2.0	5.2 ± 2.0	*P* < 0.01
Tumor depth	Limited to mucosa or submucosa	368	95	0.03
	Invades muscularis propria or deeper	2	3	
Mitotic index (/10HPF)	<2	388	96	0.26
	≤2, ≤20	3	2	
Ki-67 index (%)	≤2	383	95	0.54
	<2, ≤20	8	3	
Lymphatic vessel invasion	Absent	372	81	*P* < 0.01
	Present	19	17	
Blood vessel invasion	Absent	357	71	*P* < 0.01
	Present	34	27	

*Study population described in Table 1*.

*NETs, neuroendocrine tumors*.

**Table 6:** **Lymph node metastasis in NET G1and G2 <10 mm**.

No	Age (years)	Sex	Tumor size (mm)	Invasion	Lymphatic invasion	Blood vessel invasion	WHO 2010 classification
1	71	M	8	SM	+	+	G1
2	49	F	6	SM	−	+	G1
3	50	M	6	SM	−	+	G1
4	61	M	8	SM	+	+	G1
5	62	F	8	SM	−	+	G1
6	42	F	8	SM	+	–	G1
7	63	M	4	SM	−	+	G1
8	60	M	8	SM	−	−	G1
9	56	F	9	SM	+	−	G1

*Study population described in Table 1*.

*NETs, neuroendocrine tumors*.

## Discussion

In this study, we examined the clinicopathological association with synchronous lymph node metastasis in surgically resected rectal NET G1 and G2. Gastrointestinal NET without lymph node metastasis can be cured by local resection. Although many primary NETs themselves can be removed endoscopically, lymph node metastases can remain. Therefore, prediction of lymph node metastasis using the primary tumor is important to determine a patient’s need for surgical resection with lymph node dissection. Tumor size and tumor depth have been reported to be important for the prediction of lymph node metastasis in rectal NET (9, 10). T stage in current ENET TNM and AJCC/UICC TNM classification was determined by tumor size and depth. ENET and NCCN guidelines recommended local resection in tumors <20 mm and without muscular invasion which corresponding to pT1a and 1b (4, 5). However, our results from surgical specimens of rectal NET G1 and G2 revealed that lymphatic vessel and blood vessel invasion was the independent risk factors of lymph node metastasis. Then, 26.5% of lymph node metastasis was found in tumors smaller than 20 mm. Furthermore, 9.2% of lymph node metastases were found in tumors smaller than 10 mm, and most of these cases were positive for vascular invasion. Similar results were reported previously by Konishi et al. (11). They reported a 7% rate of lymph node metastasis in tumors smaller than 10 mm, and all cases had lymphatic vessel invasion. The JSCCR and Japan Neuroendocrine Tumor Society recommend, based on this result, local resection only for tumors smaller than 10 mm, and without muscular invasion or vascular invasion (12). There were no distinct guidelines about the indication of local resection during this study period of 2001 and 2011. Despite this, many clinicians in local institutions recommended surgical resection in tumors near 10 mm in size or cases with vascular invasion. Such a decision by local clinicians is thought to be reflected in the difference between surgically resected and locally resected cases in rectal NET <10 mm, where 9.2% of cases were positive for lymph node metastasis. Therefore, clinicians should remember that some cases <20 mm or even <10 mm in size may show lymph node metastasis in rectal NET. However, good clinical results after endoscopic resection of NET have also been reported (13). Therefore, further study with follow-up data is required to determine the optimal treatment algorithm in small rectal NET. All institutions in this study have also entered into a pathology survey, and their assessment statuses were investigated (14). In this survey, 88.6% of these institutions routinely performed immunohistochemical and histochemical staining in the diagnosis of NET, and 90.3% of these institutions routinely performed immunohistochemical and histochemical staining to assess vascular invasion. Detailed pathology evaluations were also performed by institutions entered in this study. Inter-observer differences in the assessment of vascular invasion have been reported. However, this difference seemed to be affected by the tumor size. Many NETs were found to be smaller than 10 mm in this study. Therefore, vascular invasion in rectal NET with a small size can be concordantly assessed (15, 16). Pathological analyses used in treatment algorithms should be designed to ensure objectivity in the assessment of vascular invasion in NET. In addition to the utility of describing histological features of NET from different organs, NET classification can be a predictive or prognostic factor. Our result revealed that G2 classified tumors in the WHO 2010, although not an independent risk factor did show more frequent lymph node metastasis. However, this study did not investigate clinical outcomes and recurrence, and these associations with NET classification should be investigated in the future. Similarly, this study investigated only resectable tumors and distant metastasis was not investigated. Further clinicopathological analysis will be required to investigate risk factor of metachronal distant metastasis.

This multi-institutional study also provided extensive clinicopathological information about NET of the large intestine in Japan according to NET classifications. Rectal NET has been reported to occur with higher frequency among Asian/Pacific islanders (16). In this study, we also found a very high frequency of rectal NET in the Japanese population, and tumor distributions in the large intestine are thought to be much different from that seen in Western countries (17). Next, we also found site-dependent histological variation within the large intestine in the Japanese cohort (18). Over 90% of rectal carcinoids were NET G1, and the ratio was higher than that found in the colon or appendix. This predominant incidence of G1 in rectal NET is also higher than the 40–70% range of NET in other gastrointestinal organs reported previously (19–23). Therefore, this report revealed that in addition to the tumor size or invasion, the distribution of histological grade and type is also variable among rectal, colonic, and appendiceal NET in Asian populations. Furthermore, we first reported the incidence of MANEC in the colon and rectum; MANEC were found in 1.3% of total cases, and were more frequently seen in colonic NET. Further study will be required to establish a therapeutic strategy for MANEC. We succeeded to elucidate anatomical site-dependent histological variety of NET in large intestine using uniform classification. WHO 2010 criteria can be used to understand the histological and biological variety of NET within whole body.

In conclusion, the clinicopathological features of NET were variable among the rectum, colon, and appendix, and histological variability among them clearly emerged using the NET classification. In rectal NET, our study revealed lymph node metastases even in tumors smaller than 10 mm, demonstrating that indications for surgical resection should be re-examined, especially in small NET with vascular invasion.

## Author Contributions

Conception, design of the work: MK, AO, NSaito, MI, TW, and KS. Data acquisition, analysis, and interpretation: MK, KI, NSakuyama, KK, and SK. Drafting: MK. Agreement and final approval: all authors.

## Conflict of Interest Statement

The authors declare that the research was conducted in the absence of any commercial or financial relationships that could be construed as a potential conflict of interest.
